# Effect of psychosocial interventions for depression in adults with chronic kidney disease: a systematic review and meta-analysis

**DOI:** 10.1186/s12882-023-03447-0

**Published:** 2024-01-10

**Authors:** Hui Yang, Li Qi, Dongmei Pei

**Affiliations:** Department of Health Management, Shengjing Hospital of China Medical University, No. 36, Sanhao Street, Heping District, Shenyang, 110004 China

**Keywords:** Psychotherapy, Depressive symptoms, Chronic kidney disease, Dialysis, Meta-analysis

## Abstract

**Background:**

People with chronic kidney disease (CKD) treated with dialysis are frequently affected by depression. Psychotherapy has been reported to decrease depressive symptoms in various chronic diseases and is a potential treatment option for depression. We aimed to perform a systematic review and meta-analysis to evaluate the effect of psychotherapy on depression in adults with CKD.

**Methods:**

We searched MEDLINE, Embase, Web of Science, and Cochrane for published studies up to October 31, 2023. Two investigators independently reviewed the included studies and extracted relevant data. Randomized controlled trials (RCTs) assessing the impact of interventions that provide psychological, emotional, or social support without the use of pharmacological substances on depressive symptoms in people with CKD were included and summarized. Scores on different tools for depressive assessment and quality of life were pooled.

**Results:**

A total of 19 RCTs published between 2004 and 2023 were included and analyzed. The weighted mean difference (WMD) for all included studies with regard to depression was − 2.32 (95%CI=-3.83, -0.80, *P* = 0.003). The WMD for Beck Depression Inventory (BDI) score of depression was − 3.27 (95%CI=-7.81, 1.27, *P* = 0.158) with significant heterogeneity (I^2^ = 95.1%). Significant WMD was detected for the Hospital Anxiety and Depression Scale (HADS) tool: WMD=-1.90, 95%CI=-2.91, -0.90, *P* < 0.001. The WMD for all included studies regarding quality of life was 1.21 (95%CI=-0.51, 2.93, *P* = 0.168). The WMD for Kidney Disease Quality of Life Short Form (KDQOL-SF) score was 4.55 (95%CI = 0.50, 8.60, *P* = 0.028). The WMD for SF-36 score was 0.02 (95%CI=-10.33, 10.36, *P* = 0.998). Significant difference on outcomes of S-PRT scale was observed (WMD = 2.42, 95%CI = 1.07, 3.76, *P* < 0.001).

**Conclusions:**

Psychosocial interventions probably reduce the depression level among CKD patients. Preliminary evidence suggests that psychosocial interventions might be beneficial for the quality of life in CKD patients. Our results provide medical facilities with an evidence-based basis for establishing psychosocial interventions in kidney care settings.

**Supplementary Information:**

The online version contains supplementary material available at 10.1186/s12882-023-03447-0.

## Background

Chronic kidney disease (CKD) is diagnosed in the presence of an estimated glomerular filtration rate of < 60 mL/min/1.73 m^2^ and/or elevated markers of kidney damage for at least 3 months [1]. The worldwide prevalence of CKD was as high as 9.1% in 2017 [2], and the health impacts of CKD are substantial in terms of mortality and morbidity [3]. With a high burden of somatic symptoms of CKD, impaired quality of life, and role impairment, CKD patients who require dialysis treatment were found to be in severe psychological distress [4, 5]. According to a national survey of United States (US) adults, the 12-month prevalence of depressive disorder was 10.4% [6]. Previous studies have shown that depression affects an estimated 300 million people and represents a leading cause of health-related disabilities [7]. Due to the superimposed effect of CKD, the influence of depression might be more severe among CKD patients compared with general populations. A previous meta-analysis found that 19% of CKD patients had anxiety disorders and 43% had anxiety symptoms, [8] and the literature has reported wide variability in the reported prevalence of depression in CKD patients [9, 10]. A previous systematic review and meta-analysis of observational studies showed that the summarized prevalence of depressive symptoms in patients with CKD (Stage 1–5) and transplant recipients were 26.5% and 26.6%, respectively [11].

Psychosocial interventions can be defined as interventions that provide psychological, emotional, or social support without using pharmacological substances. Psychosocial interventions may help reduce distressing symptoms, increase coping strategies, increase social connectedness, assist in strategies to address specific disease-related problems, and decrease anxiety and stress [12]. However, there is currently no uniform treatment standard and way for psychotherapy. The intensity or method, and the level of contact with individual therapists or support workers may vary. Psychosocial interventions may be especially appropriate for patients with CKD, since they avoid potential drug interactions and adverse effects of antidepressant medication. Given that depressive symptoms are associated with reduced treatment adherence, impaired functional capacity, and higher rates of hospitalization [13], several randomized controlled trials (RCTs) have been conducted to explore the association between psychosocial interventions for depression and CKD adults [14, 15].

However, the synthesis effect of comprehensive psychotherapy among CKD patients is still unclear and it is debatable whether the psychosocial interventions actually reduce the level of depression. Therefore, the meta-analysis focusing on different psychological interventions including cognitive and behavioral therapies, exercise training, or counselling is needed. In our study, we aimed to collect evidence from RCTs and investigate the effects of psychotherapy (such as cognitive and behavioral therapies, exercise training, and counselling) for depression in adults with CKD.

## Methods

### Search strategy

This systematic review and meta-analysis were performed and reported in accordance with the preferred reporting items for systematic reviews and meta-analyses (PRISMA) checklist [16]. PubMed, Embase, Web of Science, and Cochrane Central Register of Controlled Trials were used for potential studies up to October 31, 2023. A combination of Medical Subject Headings (MeSH) and text terms without language restriction were used: “kidney disease”, “renal disease”, “disease, kidney”, “diseases, kidney”, “kidney diseases”, “renal insufficiency”, “renal insufficiencies”, “kidney insufficiency”, “insufficiency, kidney”, “kidney insufficiencies”, “depression”, “depressive symptoms”, “depressive symptom”, “symptom, depressive”, “emotional depression” and “depression, emotional”. The search strategy can be found in Table S1. Reference lists of review articles, relevant studies, and clinical practice guidelines were also conducted on Google Scholar website for the previous review papers. We confirm that all methods were performed in accordance with the relevant guidelines.

### Inclusion and exclusion criteria

The inclusion were:

- Population: CKD patients aged 18 years or older with (clinical) depression;
- Intervention: psychosocial interventions (such as cognitive and behavioral therapies, exercise training, counselling and other non-pharmacological treatments) were used and assessed;
- Control: standard care or usual treatment;
- Outcome: degree of depression or quality of life;
- Study design: RCTs.

Studies were excluded if they were:

- Studies evaluating treatment for other psychiatric disorders, including bipolar affective disorder; necessary data cannot be obtained after contacting the authors of relevant studies and requesting the sharing of unpublished data; duplicate publications (summarize all studies into one ‘record’ in the review); studies published not in English.

### Study selection

Two authors independently reviewed study titles and abstracts. Both authors obtained and reviewed full-text articles of studies that were considered to be potentially relevant in order to determine their eligibility. All disagreements during study screening were resolved by a third author. Reasons for exclusion of full texts were collected.

### Data extraction

Data extraction was conducted independently by the two authors using a standardized data extraction form using Microsoft Excel (Microsoft Corp.). The type of study design, setting, country, time frame, duration of follow-up, number of participants in each group, inclusion criteria, exclusion criteria, age, sex, details of intervention, and outcomes were collected.

### Risk-of-bias and quality of studies assessment

The risk of bias and quality of the included studies were assessed with the revised Cochrane risk-of-bias tool [17]. This is a 5-domain tool including the assessment of bias arising from the randomisation process, bias due to deviations from intended interventions, bias due to deviations from intended interventions, bias in measurement of the outcome, and bias in selection of the reported result; “low concerns”, “high concerns” or “some concerns” was rated for each item in each domain, based on which overall predicted direction of bias was made for each study [17].

### Statistical analysis

An overall meta-analysis was performed if there were two or more estimates, irrespective of study design. The weighted mean difference (WMD) of scores assessed before and after intervention from each study was calculated and a random-effect model were used to combine the MDs [18]. Heterogeneity among the outcomes of included studies in this meta-analysis was evaluated using Higgins’s I^2^. Generally, I^2^ > 50% could be considered as substantial heterogeneity [19]. Statistical analyses were performed using Stata 15.0 (Stata Corporation, TX, USA).

### Publication bias assessment

Potential publication bias was evaluated using the Begg rank correlation [20] and Egger weighted regression methods [21]. The Begg and Egger tests were conducted with Stata 15.0. A two-side *P*-value of < 0.05 was considered to be significant for all analyses. The publication bias was also evaluated by funnel plots, which provide a useful graphical representation of the presence of bias in the meta-analysis. If the funnel plot is symmetrical, there is no publication bias. Otherwise, the presence of publication bias or other heterogeneity is considered.

### Sensitivity analysis

We performed sensitivity analysis to explore the robustness of all pooled effect sizes.

### Role of the funding source

The funders of the individuals working on the study had no role in study design, data collection, data analysis, data interpretation, or writing of the report.

## Results

### Literature search and characteristics of the selected studies

**Fig. 1:**
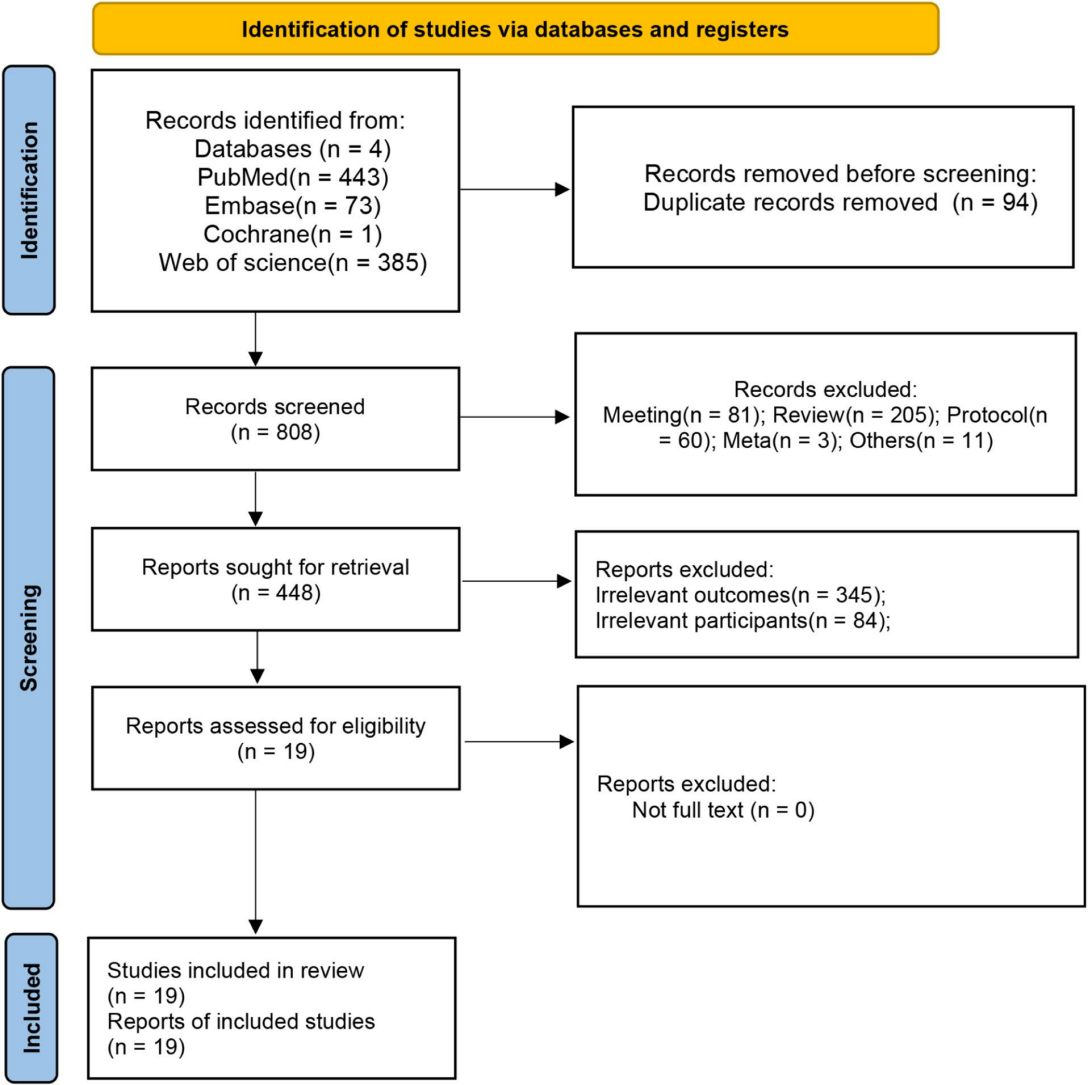
Flow chart of the study selection

As shown in Fig. 1, from the combination of four database searches, our initial search identified 902 records. After removing duplicates, 808 records remained. We then reviewed the titles, abstracts and full texts, 789 irrelevant articles were excluded. Ultimately, a total of 19 studies [14, 15, 22–38] were finally included in this meta-analysis.

### Study characteristics

The characteristics of the included studies are summarized in Table 1. All studies were RCTs. The studies were published between 2004 and 2023. The participants were from USA (*n* = 4), UK (*n* = 2), Brazil (*n* = 2), China (*n* = 5), Australia (*n* = 2), Iran (*n* = 2), Jordan (*n* = 1), and Singapore (*n* = 1). A majority of the participants were hemodialysis patients (9 studies) and comparison group accepted patients-usual care. The sample size ranged from 56 to 124. Table 2 shows tools used to assess depression and quality of life. A series of tools were used to assess depression and quality of life, including Hospital Anxiety and Depression Scale (HADS), Kidney Disease Quality of Life Short Form (KDQOL), the 36/12-item Short Form Health Survey (SF‐36/12), the physical component summary (PCS), and item Self-Perception Relationship Tool (S-PRT-28), et al. The HADS [39] is a self-rating scale consisting of two subscales, HADS-A and HADS-D, with A total of 14 items, of which 7 items rate anxiety (A) and 7 items rate depression (D). Each item was scored on a 4-point scale according to the frequency of symptoms in the last month. Each item was scored from 0 to 3, and the higher the score, the more severe the anxiety or depression symptoms. It is a very brief, easy-to-use screening measure to detect the presence of clinically significant symptoms of anxiety and is designed for use in medical populations [40]. The KDQOL [41] included two parts: kidney disease and dialysis-related quality of life and general health-related quality of life. The KDTA section consists of 43 items, each with a minimum score of 0 and a maximum score of 100, and the score in that domain is the average score of all items in the domain. The SF-36 section is divided into eight dimensions. The SF-36/12 includes eight dimensions, and the total score of the eight dimensions is the total score of the scale, which is more sensitive to change and is often used to assess the effect of treatment. The PCS [42] is derived from a selection of responses to yield a single score between 0 and 100, with a mean of 50 and standard deviation (SD) of 10 in population studies, which reflects “physical health.” The PCS has robust psychometric properties, and has been used in a large number of trials in many different conditions.

**Table 1:** Characteristics of included studies

**Study included**	**Country**	**Randomized method**	**Intervention method**	**Intervention group**	**Comparison group**	**Drugs**	**Intervention group**			**Comparison group**			**Follow up**
							**age**	**% males**	**n**	**age**	**% males**	**n**	
Sharp et al., 2005 [29]	UK	Computer	Educational, cognitive, and behavioral strategies	Hemodialysis patients	Patients-Usual care	Not report	56.05 (12.73)	62	29	52.52 (12.70)	84	27	None
Song et al., 2009 [30]	USA	Randomized number table	Sharing Patients’ Illness Representations to Increase Trust (SPIRIT)	Hemodialysis patients	Patients-Usual care	Not report	58.31 (11.8)	65	29	57.55 (12.2)	48	29	12 weeks
Duarte et al., 2009 [25]	Brazil	Randomized number table	Cognitive–behavioral therapy (CBT)	Hemodialysis patients	Patients-Usual care	Not report	52 (16)	37	46	54 (13)	46	44	6 months
Rodriguez et al., 2011 [15]	USA	Randomized number table	Quality of life therapy (QOLT),	Chronic kidney (creatinine clearance <15 mL/min)	Standard care (SC)	Not report	53 (11)	54	22	52 (12)	40	20	12 weeks
Moattari et al., 2012 [28]	Iran	Randomized number table	Empowerment intervention	Hemodialysis patients	Patients-Usual care	Not report	38 (11)	60	25	37 (11)	70	25	6 weeks
Hare et al., 2014 [26]	UK	Randomized number table	Educational, cognitive, and behavioral strategies	Peritoneal dialysis patients	Patients-Usual care	Not report	60	100	8	60	99	7	6 weeks
Chan et al., [22]	China	Computer	Enhanced psychosocial support	Chronic kidney (creatinine clearance <15 mL/min)	Patients-Usual care	Not report	82 (4)	57	14	81 (6)	47	15	NA
Tang et.al., 2017 [43]	China	Computer	Individualized exercise program	Hemodialysis patients	Treatment as usual	Not report	46.26 (15.61)	66.67	42	43.90 (12.44)	54.76	42	6 months
Jenkins et.al., 2020 [27]	Australia	Computer	Kidney Optimal Health Program (KOHP)	stage 4 or 5 chronic kidney disease	Patients-Usual care	Not report	60.8 (10.19)	59.3	27	59.78 (13.19)	46.7	30	12 months
Morais et.al., 2020 [14]	Brazil	Cluster randomization	Practice of presenting comedy movies	Hemodialysis patients	Patients-Usual care	Not report	61.7 (14.6)	74.3	35	61.7 (13.2)	65.4	26	6 weeks
Dingwall et.al., 2021 [24]	Australia	Computer	Immediate AIMhi Stay Strong App treatment	End stage kidney disease	Treatment as usual	Not report	55 (10.6)	25.8	16	57.0 (8.2)	27.3	9	3-month
Tsay et.al., 2004 [37]	China	Computer	Empowerment program	End-stage renal disease	Patients-Usual care	Not report	NA	36	25	NA	44	25	6 weeks
Tsay et.al., 2005 [38]	China	Computer	Adaptation training programme	End-stage renal disease	Patients-Usual care	Not report	NA	46.7	30	NA	48.2	27	3-month
Tsai et.al., 2015 [36]	China	Computer	Nurse-led breathing training programme	Chronic kidney (creatinine clearance <15 mL/min)	Patients-Usual care	Not report	64.94 (9.51)	50	32	61.08 (11.18)	48	25	6 weeks
Babamohamadi et.al., 2017 [32]	Iran	Randomized number table	Holy Qur’an Recitation	Hemodialysis patients	Patients-Usual care	Not report	50.2 (12.9)	51.9	27	56.4 (8.9)	63	27	1 month
Saraireh et.al., 2018 [31]	Jordanian	Randomized number table	Cognitive–behavioral therapy (CBT)	Renal dialysis patients	Psychoeducation	Not report	52 (10.7)	NA	54	53.4 (8.0)	NA	51	3-month
Mehrotra et.al., 2019 [35]	USA	Randomized number table	Cognitive–behavioral therapy (CBT)	Hemodialysis patients	Patients-Usual care	Sertraline 25mg/day	50 (13)	55	60	53 (12)	58	60	12 weeks
Látos et.al., 2022 [34]	USA	Randomized number table	The Four‑Step Positive Psychology Intervention	End stage kidney disease	Patients-Usual care	Not report	49.2 (9.89)	NA	20	49.5 (10.62)	NA	20	6 months
Chen et.al., 2023 [33]	Singapore	Computer	Psychoeducational intervention	End stage kidney disease	Patients-Usual care	Not report	59.66 (12.37)	56.9	65	62.02 (13.68)	59.3	59	6 months

*Abbrevation*: *NA* Not available

**Table 2:** Tools used to assess depression and quality of life

**Study included**	**Tools**
**Depression**	
Sharp et al., 2005 [29]	HADS
Rodrigue et al., 2011 [15]	Combined
Duarte et al., 2009 [25]	Combined
Hare et al., 2014 [26]	HADS
Chan et al., 2015 [22]	HADS
Valsaraj et.al., 2016 [44]	HADS
Tang et.al., 2017 [43]	HADS
Dingwall et.al., 2021 [24]	K10
Jenkins et.al., 2020 [27]	HADS and GSE
Tsay et.al., 2004 [37]	BDI
Tsay et.al., 2005 [38]	BDI
Tsai et.al., 2015 [36]	BDI-II
Babamohamadi et.al., 2017 [32]	BDI-II
Saraireh et.al., 2018 [31]	HADS
Mehrotra et.al., 2019 [35]	BDI-II
Látos et.al., 2022 [34]	BDI
Chen et.al., 2023 [33]	HADS
**Quality of Life**	
Sharp et al., 2005 [29]	S-PRT
Duarte et al., 2009 [25]	Combined
Song et al., 2009 [30]	S-PRT
Cukor et al., 2014 [23]	KDQOL-SF
Hare et al., 2014 [26]	SF-36
Chan et al., 2015 [22]	KDQOL-SF
Tang et.al., 2017 [43]	SF‐12, PCS
Rodrigue et al., 2011 [15]	SF-36
Moattari et al., 2012 [28]	Combined
Morais et.al., 2020 [14]	KDQOL
Jenkins et.al., 2020 [27]	SF12
Jenkins et.al., 2020 [27]	KDQoL-KDCS
Dingwall et.al., 2021 [24]	PHQ-9
Tsay et.al., 2005 [38]	SF-36
Tsai et.al., 2015 [36]	SF-36
Mehrotra et.al., 2019 [35]	Combined
Chen et.al., 2023 [33]	KDQOL-SF12

*Abbreviations*: *HADS* Hospital Anxiety and Depression Scale, *KDQOL* Kidney Disease Quality of Life Short Form, *SF‐36/12* the 36/12‐item Short Form Health Survey, *PCS* the physical component summary, *S-PRT-28* item Self-Perception Relationship Tool

### Risk of bias and quality of studies

**Fig. 2:**
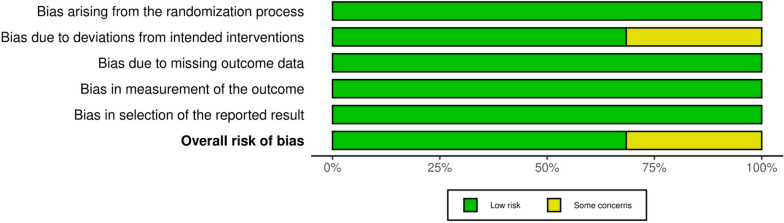
Overall risk of bias of the meta-analysis

The overall risk of bias and the quality of the included RCTs were acceptable (Fig. 2). Most studies used a randomized and blinded method for including the participants. Risk of bias assessment of included studies was shown in Figure S1, and the GRADE level of evidence was presented in Table S2.

### Outcome of depression

**Fig. 3:**
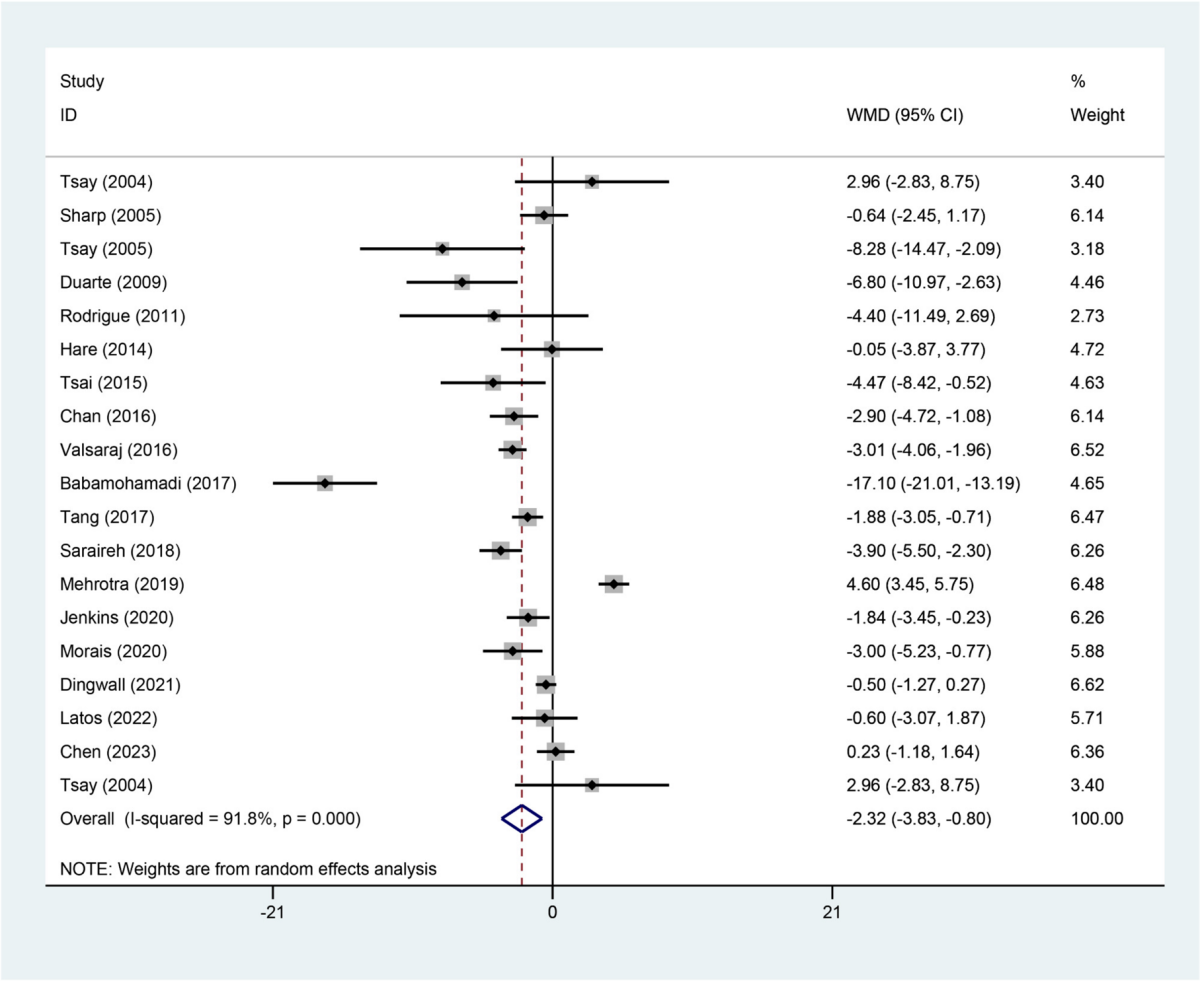
Forest plot of changes of depression

A total of 19 studies assessed baseline and follow-up depression scores. The WMD for all included studies was − 2.32 (95%CI=-3.83, -0.80, *P* = 0.003) (Fig. 3). The WMD for BDI score of depression was − 3.27 (95%CI=-7.81, 1.27, *P* = 0.158) with significant heterogeneity (I^2^ = 95.1%) (Figure S2). Significant WMD was detected for the HADS scale: WMD=-1.90, 95%CI=-2.91, -0.90, *P* < 0.001 (Figure S2). The WMD for HSCL and K10 were − 4.40 (95%CI=-11.49, 2.69, *P* = 0.224) and − 0.50 (95%CI=-1.27, 0.27, *P* = 0.205), respectively (Figure S2).

### Outcome of quality of life

**Fig. 4:**
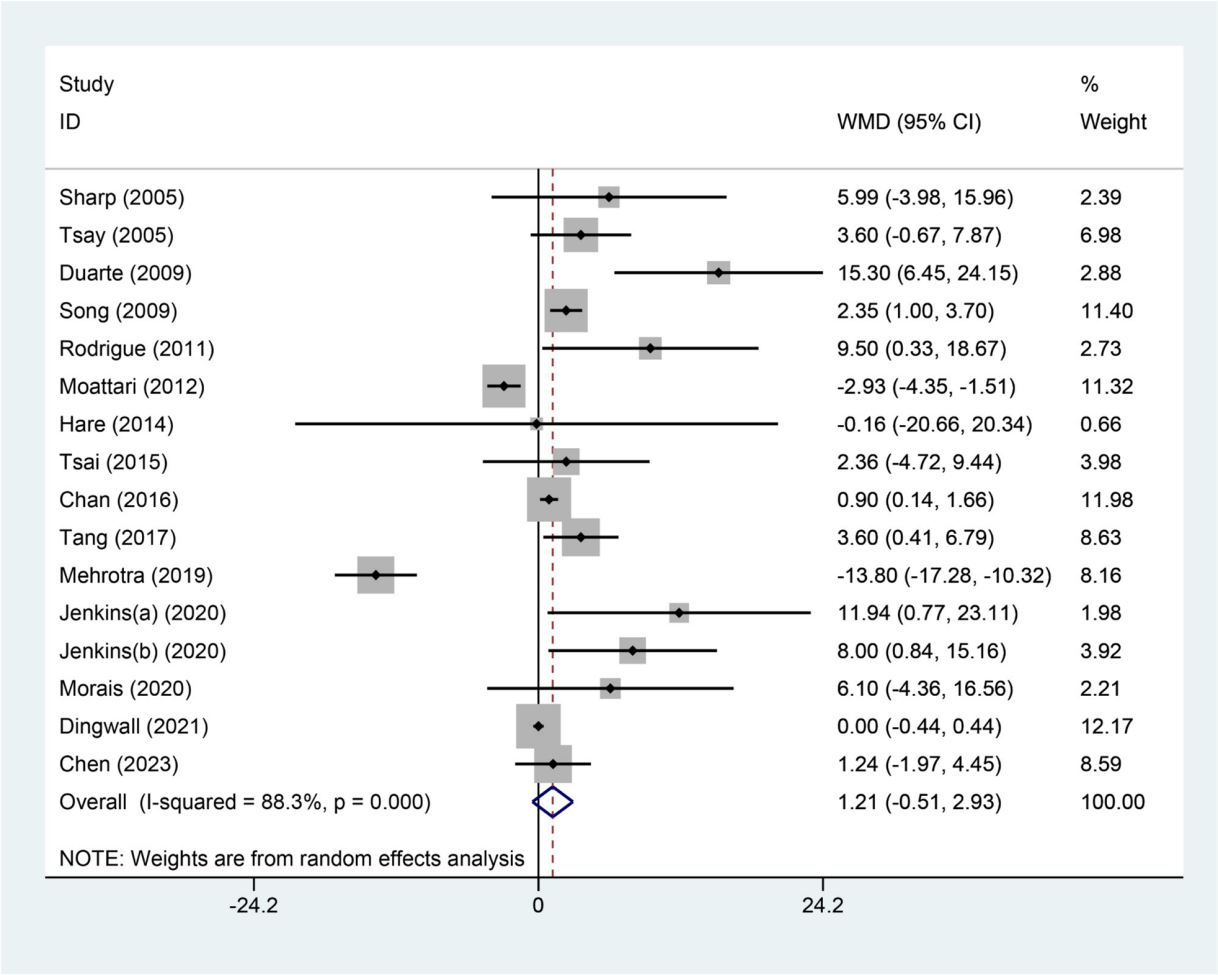
Forest plot of changes of quality of life

 Sixteen included studies reported results on the assessment of quality of life at baseline and follow-up. The WMD for all included studies was 1.21 (95%CI=-0.51, 2.93, *P* = 0.168) (Fig. 4). The WMD for KDQOL-SF score was 4.55 (95%CI = 0.50, 8.60, *P* = 0.028) (Figure S3). The WMD for SF-36 score was 0.02 (95%CI=-10.33, 10.36, *P* = 0.998) (Figure S3). Significant difference on outcomes of S-PRT scale was observed (WMD = 2.42, 95%CI = 1.07, 3.76, *P* < 0.001) (Figure S3). There was only one study reported outcomes for each scale including KDQOL-KDCS, PHQ-9, QoL, and SF-12, the respective WMD were 8.00 (95%CI = 0.84, 15.16, *P* = 0.028), 0 (95%CI=-0.44, 0.44, *P* = 1.000), 0.02 (95%CI=-10.33, 10.36, *P* = 0.998), -2.93 (95%CI=-4.35, -1.51, *P* < 0.001), and 3.60 (95%CI = 0.41, 6.79, *P* = 0.027) (Figure S3).

### Publication bias

Because only 2 studies were included in the analysis of SPRT, Egger’s test could not performed. There was no statistically significant publication bias according to both Begg’s test (*P* > 0.05) and Egger’s test (*P* > 0.05) in the analyses of other parameters (Figures S4-S8) (Table S3). Nevertheless, funnel plots for pooled results of included studies on changes of BDI, KDQOL-SF, and SF-36 are graphically asymmetrical, indicating potential bias in the included studies despite of the insignificant results on Begg’s test and Egger’s test.

### Sensitivity analysis

We conducted a sensitive analysis to assess the dependability and coherence of the final outcome. Our goal was to test the robustness of our conclusions by systematically omitting one study at a time and re-evaluating the combined effect size of the remaining dataset. This meticulous sensitivity assessment method confirmed that the overall results remained stable even with the removal of any specific study. Therefore, it is evident that no single study had a disproportionate impact on the overall results (Figures S9-S13).

## Discussion

In our current study, we systematically reviewed and summarized the effect of psychotherapy on depression in adults with CKD. In our study, by pooling 19 articles, psychotherapy could effectively remit depression for CKD and significantly improve the quality of life of people with CKD.

Depression is the most common psychological problem in patients undergoing dialysis and about 25% of dialysis patients were found major depression [11, 45, 46], which may lead to lower adherence to dialysis prescriptions, lower health-related quality of life, and even poorer clinical outcomes [47–50]. Depression was reported to be caused by medications, reduction of physical function, or restriction of daily dietary [51]. Without an optimal screening tool for the assessment of depression, several tools including the Beck Depression Inventory (BDI), Patient Health Questionnaire, and Center for Epidemiologic Studies Depression Scale are widely used for the measurement of depression in CKD people.

Psychosocial interventions, which include counselling, social group support, cognitive-behavioral therapy, relaxation or visualization techniques, exercise, education, or individual social support including by telephone, are defined as interventions that can provide psychological, emotional, or social support without using pharmacological substances and have been demonstrated to be effective for people with depression [52]. A previous meta-analysis of the effects of psychosocial interventions on social functioning in depression and schizophrenia among general population found that psychosocial interventions delivered in outpatients and primary care settings are effective in improving social functioning in people with depression and should be incorporated into efforts to scale up services [52]. In our study, we also found a positive effect of psychosocial interventions on CKD. Of note, in all included studies ot the current meta-analysis, patients in the control group (standard care or usual treatment) also received treatment, the baseline treatment for intervention group and control were equal except for the psychosocial approaches used in the group. Subsequently, results of this study highlighted the significance of specialized psycosocial interventions and certified mental health professionals in the CKD treating team. Very few studies have assessed the effect of psychosocial interventions on people with CKD and few meta‐analyses have been published before. Our findings are consistent with the findings of previous meta‐analysis of published RCTs evaluating psychosocial interventions for anxiety symptoms in individuals with CKD [53]. Our study highlighted what constitutes a psychosocial intervention on CKD, of which, “psychosocial” was widely used to describe the interventions as behavioral, educational, psychological, or social. At the same time, complementary therapies such as physical and mental intervention also play an important role in patients with CKD and dialysis. Chu et al. found that music and psychotherapy reduced anxiety symptoms by 8.06–43.5% and 36.1–41.1%, respectively, and psychotherapy reduced depressive symptoms by 56.8% [53]. While we also found there was significant heterogeneity across the included RCTs and the potential sources of the heterogeneity could be the type of interventions, follow-up duration, or baseline characteristics of CKD patients. These potential heterogeneity sources could not fully taken into account in our study owing to the limited number of studies in each subgroup under different assessment tools. Meanwhile, each included study had unique research background, such as the healthcare policy, cultural factors, and study country, which lead to an inevitable heterogeneity of our study.

Given the lack of consistency in how psychosocial interventions were defined, our findings suggest that psychosocial interventions should be defined in the context of both psychological and social components. However, due to the time requirements of dialysis for CKD cases and the feeling of fatigue after dialysis, many patients may be reluctant to participate in a time-intensive psychosocial intervention. That would be a major concern that impedes the practice of psychosocial interventions. A previous meta-analysis [54] focusing on psychotherapies across general population in different age groups reported the small but statistically significant effect sizes of psychotherapies in general population. According to the results of our study, there were significant in decreasing the levels of depression and improvement in quality of life after psychotherapy intervention compared with baseline. However, the clinically relevant could be limited due to the WMDs were also small. In the clinical application process, the significant positive effect might not be observed straightly. Compared with conventional treatment, psychological intervention did have a positive effect on CKD patients, even if it was statistically significant. Future studies could carry out psychological interventions in CKD patients with different disease stages to discover that in the practical clinical applications the intervention was the most effective among which subgroups of CKD patients. In addition, it is not possible to definitively establish the impact of psychosocial interventions on major depression, anxiety, withdrawal from dialysis, or death from any cause. The potential adverse events of treatment are largely unknown. Feasible, effective, and acceptable interventions are needed and may be sustainable for implementation within clinical settings.

There were several limitations in our study. First, several important individual pieces of information were not provided, thus we couldn’t perform a more accurate analysis of clinical characteristics. Meanwhile, due to insufficient information, we cannot deploy more subgroup analyses, especially on medication use, more relevant RCTs are warranted to investigate the impact of medication on the effects of psychosocial interventions for depression in CKD adults. Second, although a comprehensive search strategy was independently performed by 2 investigators in 4 databases with cross reviewing, we cannot guarantee that all relevant studies have been included in our analysis. Third, the funnel plots for included studies on changes of BDI, KDQOL-SF, and SF-36 are indeed asymmetrical, but no publication bias between studies was shown based on the results of Begg’s test and Egger’s test, which suggest the limitations to these methods. The asymmetry of the funnel plot may be due to other source of heterogeneity across enrolled studies, which is proved by the high I^2^ values. Fourth, depressive outcomes and quality of life were assessed using various tools. Depression was deemed as a continuous outcome either as major (or severe) depression or depression (end of treatment) and the severity of depressive symptoms was assessed as a dichotomous outcome. Standardization of outcome reporting in future psychosocial intervention trials is therefore needed.

## Conclusions

Our study suggests that psychosocial interventions are promising for improving psychological well-being in adults with CKD. Psychosocial interventions, such as cognitive and behavioral therapies, exercise training, and counselling, may reduce depression and improve quality of life in these patients. In future, researchers investigating psychosocial treatments should consider the use of standardized interventions.

### Supplementary Information

**Additional file 1:** **Table S1. **Search strategy of PubMed, Embase, Cochrane Library and Web of science.

**Additional file 2:** **Table S2. **GRADE level of evidence.

**Additional file 3:** **Table S3. **Publication bias of summarized outcomes.

**Additional file 4: Figure S1.** Risk of bias assessment of included studies using the revised Cochrane risk of bias tool. **Figure S2.** Forest plot of subgroup analyses on changes of depression. **Figure S3.** Forest plot of changes of subgroup analyses on quality of life. **Figure S4.** Funnel plot for pooled results of included studies on changes of BDI. **Figure S5.** Funnel plot for pooled results of included studies on changes of HADS. **Figure S6.** Funnel plot for pooled results of included studies on changes of KDQOL-SF. **Figure S7.** Funnel plot for pooled results of included studies on changes of SF-36. **Figure S8.** Funnel plot for pooled results of included studies on changes of SPRT. **Figure S9.** Results of sensitivity analysis for pooled results of included studies on changes of BDI. **Figure S10.** Results of sensitivity analysis for pooled results of included studies on changes of HADS. **Figure S11.** Results of sensitivity analysis for pooled results of included studies on changes of KDQOL-SF. **Figure S12.** Results of sensitivity analysis for pooled results of included studies on changes of SF-36. **Figure S13.** Results of sensitivity analysis for pooled results of included studies on changes of SPRT.

## Data Availability

All data generated or analysed during this study are included in this published article.

## Abbreviations

RCTs: Randomized controlled trials
SD: Standard deviation
WMD: Weighted mean difference
CKD: Chronic kidney disease
MeSH: Medical Subject Heading
HADS: Hospital Anxiety and Depression Scale
KDQOL: Kidney Disease Quality of Life Short Form
BDI: Beck Depression Inventory
