# Supplementary material for: Effect of psychosocial interventions for depression in adults with chronic kidney disease: a systematic review and meta-analysis
Source: BMC Nephrol. 2024 Jan 10;25:17. doi: 10.1186/s12882-023-03447-0 (PMC10782786; doi:10.1186/s12882-023-03447-0)
Supplement: Supplementary file 1 — Additional file 1: Table S1. Search strategy of PubMed, Embase, Cochrane Library and Web of science. [file 12882_2023_3447_MOESM1_ESM.docx]

**Table S1 Search strategy of PubMed, Embase, Cochrane Library and Web of science**

Search date: 2023/10/31

| **PubMed** |  | **Search strategy** | | **Numbers** |
| --- | --- | --- | --- | --- |
| **#1** | **"Renal Insufficiency, Chronic"[Mesh]** | |  | **135,503** |
| **#2** | **"Renal Insufficiency"[Mesh]** | |  | **204,579** |
| **#3** | **"Depressive Disorder"[Mesh]** | |  | **122,690** |
| **#4** | **"Depression"[Mesh]** | |  | **152,644** |
| **#5** | **((((((((((((((((((((((Chronic Renal Insufficiencies) OR (Chronic Renal Insufficiency)) OR (Chronic Kidney Insufficiency)) OR (Chronic Kidney Insufficiencies)) OR (Chronic Kidney Diseases)) OR (Chronic Kidney Disease)) OR (Chronic Renal Diseases)) OR (Chronic Renal Disease)) OR (Renal Insufficiencies)) OR (Kidney Insufficiency)) OR (Kidney Insufficiencies)) OR (Kidney Failure)) OR (Kidney Failures)) OR (Renal Failure)) OR (Renal Failures)) OR (chronic kidney disorder)) OR (chronic nephropathy)) OR (chronic renal failure)) OR (kidney chronic failure)) OR (chronic kidney failure)) OR (maternal kidney failure)) OR (renal insufficiency)) OR (terminal kidney failure)** | |  | **398,496** |
| **#6** | **(((((((((((((((((((((((((((Depressive Symptoms) OR (Depressive Symptom)) OR (Emotional Depression)) OR (Depress)) OR (central depression)) OR (clinical depression)) OR (depressive disease)) OR (depressive disorder)) OR (depressive episode)) OR (depressive illness)) OR (depressive personality disorder)) OR (depressive state)) OR (depressive syndrome)) OR (mental depression)) OR (parental depression)) OR (depression)) OR (Depressive Disorders)) OR (Depressive Neuroses)) OR (Depressive Neurosis)) OR (Endogenous Depression)) OR (Endogenous Depressions)) OR (Depressive Syndromes)) OR (Neurotic Depression)) OR (Neurotic Depressions)) OR (Melancholia)) OR (Melancholias)) OR (Unipolar Depression)) OR (Unipolar Depressions)** | |  | **637,451** |
| **#7** | **"Psychosocial Intervention"[Mesh]** | |  | **986** |
| **#8** | **((((((((Psychosocial Interventions) OR (Psychological Intervention)) OR (Psychological Interventions)) OR (psycho-social intervention)) OR (psycho-social therapy)) OR (psycho-social treatment)) OR (psychosocial therapy)) OR (psychosocial treatment)) OR (psychosocial intervention)** | |  | **185,125** |
| **#9** | **(#1 OR #2 OR #5) AND (#3 OR #4 OR #6) AND (#7 OR #8)** | |  | **443** |

| **Embase** | |  | **Search strategy** | | **Numbers** |
| --- | --- | --- | --- | --- | --- |
| **#1** | **'kidney failure'/exp** | | |  | **558938** |
| **#2** | **'chronic kidney failure'/exp** | | |  | **208931** |
| **#3** | **'chronic renal insufficiencies':ti,ab,kw OR 'chronic renal insufficiency':ti,ab,kw OR 'chronic kidney insufficiency':ti,ab,kw OR 'chronic kidney insufficiencies':ti,ab,kw OR 'chronic kidney diseases':ti,ab,kw OR 'chronic kidney disease':ti,ab,kw OR 'chronic renal diseases':ti,ab,kw OR 'chronic renal disease':ti,ab,kw OR 'renal insufficiencies':ti,ab,kw OR 'kidney insufficiency':ti,ab,kw OR 'kidney insufficiencies':ti,ab,kw OR 'kidney failure':ti,ab,kw OR 'kidney failures':ti,ab,kw OR 'renal failure':ti,ab,kw OR 'renal failures':ti,ab,kw OR 'chronic kidney disorder':ti,ab,kw OR 'chronic nephropathy':ti,ab,kw OR 'chronic renal failure':ti,ab,kw OR 'kidney chronic failure':ti,ab,kw OR 'chronic kidney failure':ti,ab,kw OR 'maternal kidney failure':ti,ab,kw OR 'renal insufficiency':ti,ab,kw OR 'terminal kidney failure':ti,ab,kw** | | |  | **318009** |
| **#4** | **'depression'/exp** | | |  | **654198** |
| **#5** | **'depressive symptoms':ti,ab,kw OR 'depressive symptom':ti,ab,kw OR 'emotional depression':ti,ab,kw OR 'depress':ti,ab,kw OR 'central depression':ti,ab,kw OR 'clinical depression':ti,ab,kw OR 'depressive disease':ti,ab,kw OR 'depressive disorder':ti,ab,kw OR 'depressive episode':ti,ab,kw OR 'depressive illness':ti,ab,kw OR 'depressive personality disorder':ti,ab,kw OR 'depressive state':ti,ab,kw OR 'depressive syndrome':ti,ab,kw OR 'mental depression':ti,ab,kw OR 'parental depression':ti,ab,kw OR 'depression':ti,ab,kw OR 'depressive disorders':ti,ab,kw OR 'depressive neuroses':ti,ab,kw OR 'depressive neurosis':ti,ab,kw OR 'endogenous depression':ti,ab,kw OR 'endogenous depressions':ti,ab,kw OR 'depressive syndromes':ti,ab,kw OR 'neurotic depression':ti,ab,kw OR 'neurotic depressions':ti,ab,kw OR 'melancholia':ti,ab,kw OR 'melancholias':ti,ab,kw OR 'unipolar depression':ti,ab,kw OR 'unipolar depressions':ti,ab,kw** | | |  | **679548** |
| **#6** | **'psychosocial intervention'/exp** | | |  | **2127** |
| **#7** | **'psychosocial interventions':ti,ab,kw OR 'psychological intervention':ti,ab,kw OR 'psychological interventions':ti,ab,kw OR 'psycho-social intervention':ti,ab,kw OR 'psycho-social therapy':ti,ab,kw OR 'psycho-social treatment':ti,ab,kw OR 'psychosocial therapy':ti,ab,kw OR 'psychosocial treatment':ti,ab,kw OR 'psychosocial intervention':ti,ab,kw** | | |  | **24392** |
| **#8** | **(#1 OR #2 OR #3) AND (#4 OR #5) AND (#6 OR #7)** | | |  | **73** |

| **Cochrane** | |  | **Search strategy** | | **Numbers** |
| --- | --- | --- | --- | --- | --- |
| #1 | MeSH descriptor: [Renal Insufficiency, Chronic] explode all trees | | |  | 8678 |
| #2 | MeSH descriptor: [Renal Insufficiency] explode all trees | | |  | 11725 |
| #3 | MeSH descriptor: [Depressive Disorder] explode all trees | | |  | 15332 |
| #4 | MeSH descriptor: [Depression] explode all trees | | |  | 18751 |
| #5 | MeSH descriptor: [Psychosocial Intervention] explode all trees | | |  | 232 |
| #6 | (Chronic Renal Insufficiencies):ti,ab,kw OR (Chronic Renal Insufficiency):ti,ab,kw OR (Chronic Kidney Insufficiency):ti,ab,kw OR (Chronic Kidney Insufficiencies):ti,ab,kw OR (Chronic Kidney Diseases):ti,ab,kw OR (Chronic Kidney Disease):ti,ab,kw OR (Chronic Renal Diseases):ti,ab,kw OR (Chronic Renal Disease):ti,ab,kw OR (Renal Insufficiencies):ti,ab,kw OR (Kidney Insufficiency):ti,ab,kw OR (Kidney Insufficiencies):ti,ab,kw OR (Kidney Failure):ti,ab,kw OR (Kidney Failures):ti,ab,kw OR (Renal Failure):ti,ab,kw OR (Renal Failures):ti,ab,kw OR (chronic kidney disorder):ti,ab,kw OR (chronic nephropathy):ti,ab,kw OR (chronic renal failure):ti,ab,kw OR (kidney chronic failure):ti,ab,kw OR (chronic kidney failure):ti,ab,kw OR (maternal kidney failure):ti,ab,kw OR (renal insufficiency):ti,ab,kw OR (terminal kidney failure):ti,ab,kw | | |  | 40296 |
| #7 | (Depressive Symptoms):ti,ab,kw OR (Depressive Symptom):ti,ab,kw OR (Emotional Depression):ti,ab,kw OR (Depress):ti,ab,kw OR (central depression):ti,ab,kw OR (clinical depression):ti,ab,kw OR (depressive disease):ti,ab,kw OR (depressive disorder):ti,ab,kw OR (depressive episode):ti,ab,kw OR (depressive illness):ti,ab,kw OR (depressive personality disorder):ti,ab,kw OR (depressive state):ti,ab,kw OR (depressive syndrome):ti,ab,kw OR (mental depression):ti,ab,kw OR (parental depression):ti,ab,kw OR (depression):ti,ab,kw OR (Depressive Disorders):ti,ab,kw OR (Depressive Neuroses):ti,ab,kw OR (Depressive Neurosis):ti,ab,kw OR (Endogenous Depression):ti,ab,kw OR (Endogenous Depressions):ti,ab,kw OR (Depressive Syndromes):ti,ab,kw OR (Neurotic Depression):ti,ab,kw OR (Neurotic Depressions):ti,ab,kw OR (Melancholia):ti,ab,kw OR (Melancholias):ti,ab,kw OR (Unipolar Depression):ti,ab,kw OR (Unipolar Depressions):ti,ab,kw | | |  | 105083 |
| #8 | (Psychosocial Interventions):ti,ab,kw OR (Psychological Intervention):ti,ab,kw OR (Psychological Interventions):ti,ab,kw OR (psycho-social intervention):ti,ab,kw OR (psycho-social therapy):ti,ab,kw OR (psycho-social treatment):ti,ab,kw OR (psychosocial therapy):ti,ab,kw OR (psychosocial treatment):ti,ab,kw OR (psychosocial intervention):ti,ab,kw | | |  | 47776 |
| #9 | (#1 or #2 or #6) and (#3 or #4 or #7) and (#5 and #8) | | |  | 1 |

| **Web of sci** | | **Search strategy** | | **Numbers** | |  |
| --- | --- | --- | --- | --- | --- | --- |
| **#1** | **(((((((((((((((((((((TS=(Chronic Renal Insufficiencies) OR TS=(Chronic Renal Insufficiency)) OR TS=(Chronic Kidney Insufficiency)) OR TS=(Chronic Kidney Insufficiencies)) OR TS=(Chronic Kidney Diseases)) OR TS=(Chronic Kidney Disease)) OR TS=(Chronic Renal Diseases)) OR TS=(Chronic Renal Disease)) OR TS=(Renal Insufficiencies)) OR TS=(Kidney Insufficiency)) OR TS=(Kidney Insufficiencies)) OR TS=(Kidney Failure)) OR TS=(Kidney Failures)) OR TS=(Renal Failure)) OR TS=(Renal Failures)) OR TS=(chronic kidney disorder)) OR TS=(chronic nephropathy)) OR TS=(chronic renal failure)) OR TS=(kidney chronic failure)) OR TS=(chronic kidney failure)) OR TS=(maternal kidney failure)) OR TS=(renal insufficiency)) OR TS=(terminal kidney failure)** | |  | | **302997** | |
| **#2** | **((((((((((((((((((((((((((TS=(Depressive Symptoms) OR TS=(Depressive Symptom)) OR TS=(Emotional Depression)) OR TS=(Depress)) OR TS=(central depression)) OR TS=(clinical depression)) OR TS=(depressive disease)) OR TS=(depressive disorder)) OR TS=(depressive episode)) OR TS=(depressive illness)) OR TS=(depressive personality disorder)) OR TS=(depressive state)) OR TS=(depressive syndrome)) OR TS=(mental depression)) OR TS=(parental depression)) OR TS=(depression)) OR TS=(Depressive Disorders)) OR TS=(Depressive Neuroses)) OR TS=(Depressive Neurosis)) OR TS=(Endogenous Depression)) OR TS=(Endogenous Depressions)) OR TS=(Depressive Syndromes)) OR TS=(Neurotic Depression)) OR TS=(Neurotic Depressions)) OR TS=(Melancholia)) OR TS=(Melancholias)) OR TS=(Unipolar Depression)) OR TS=(Unipolar Depressions)** | |  | | **772291** | |
| **#3** | **(((((((TS=(Psychosocial Interventions) OR TS=(Psychological Intervention)) OR TS=(Psychological Interventions)) OR TS=(psycho-social intervention)) OR TS=(psycho-social therapy)) OR TS=(psycho-social treatment)) OR TS=(psychosocial therapy)) OR TS=(psychosocial treatment)) OR TS=(psychosocial intervention)** | |  | | **142481** | |
| **#4** | **#3 AND #2 AND #1** | |  | | **385** | |
