# Supplementary material for: Effect of psychosocial interventions for depression in adults with chronic kidney disease: a systematic review and meta-analysis
Source: BMC Nephrol. 2024 Jan 10;25:17. doi: 10.1186/s12882-023-03447-0 (PMC10782786; doi:10.1186/s12882-023-03447-0)
Supplement: Supplementary file 2 — Additional file 2: Table S2. GRADE level of evidence. [file 12882_2023_3447_MOESM2_ESM.docx]

**Table S2 GRADE level of evidence**

| **Outcome** | **Risk of bias** | **Inconsistency** | **Indirectness** | **Imprecision** | **Overall quality of evidence** |
| --- | --- | --- | --- | --- | --- |
| **depression** | | | | | |
| BDI | | | | | |
|  | Low | Moderate | Not serious | Not serious | Low |
| HADS | | | | | |
|  | Low | Moderate | Not serious | Not serious | Low |
| **quality of life** | | | | | |
| KDQOL-SF | | | | | |
|  | Low | Serious | Not serious | Not serious | Low |
| SF-36 | | | | | |
|  | Low | Serious | Not serious | Not serious | Low |
| SPRT | | | | | |
|  | Low | Serious | Not serious | Not serious | Low |
