# Supplementary material for: Effect of psychosocial interventions for depression in adults with chronic kidney disease: a systematic review and meta-analysis
Source: BMC Nephrol. 2024 Jan 10;25:17. doi: 10.1186/s12882-023-03447-0 (PMC10782786; doi:10.1186/s12882-023-03447-0)
Supplement: Supplementary file 3 — Additional file 3: Table S3. Publication bias of summarized outcomes. [file 12882_2023_3447_MOESM3_ESM.docx]

**Table S3. Publication bias of summarized outcomes**

Depression

| **Outcomes** | **Publication bias** | |
| --- | --- | --- |
|  | **Begg (*P* value)** | **Egger (*P* value)** |
| BDI | 0.917 | 0.050 |
| HADS | 0.621 | 0.504 |

Quality of life

| **Outcomes** | **Publication bias** | |
| --- | --- | --- |
|  | **Begg (*P* value)** | **Egger (*P* value)** |
| KDQOL-SF | 0.142 | 0.073 |
| SF-36 | 1.000 | 0.382 |
| SPRT | 0.317 | - |
