# Supplementary material for: Effect of psychosocial interventions for depression in adults with chronic kidney disease: a systematic review and meta-analysis
Source: BMC Nephrol. 2024 Jan 10;25:17. doi: 10.1186/s12882-023-03447-0 (PMC10782786; doi:10.1186/s12882-023-03447-0)
Supplement: Supplementary file 4 — Additional file 4: Figure S1. Risk of bias assessment of included studies using the revised Cochrane risk of bias tool. Figure S2. Forest plot of subgroup analyses on changes of depression. Figure S3. Forest plot of changes of subgroup analyses on quality of life. Figure S4. Funnel plot for pooled results of included studies on changes of BDI. Figure S5. Funnel plot for pooled results of included studies on changes of HADS. Figure S6. Funnel plot for pooled results of included studies on changes of KDQOL-SF. Figure S7. Funnel plot for pooled results of included studies on changes of SF-36. Figure S8. Funnel plot for pooled results of included studies on changes of SPRT. Figure S9. Results of sensitivity analysis for pooled results of included studies on changes of BDI. Figure S10. Results of sensitivity analysis for pooled results of included studies on changes of HADS. Figure S11. Results of sensitivity analysis for pooled results of included studies on changes of KDQOL-SF. Figure S12. Results of sensitivity analysis for pooled results of included studies on changes of SF-36. Figure S13. Results of sensitivity analysis for pooled results of included studies on changes of SPRT. [file 12882_2023_3447_MOESM4_ESM.docx]

**
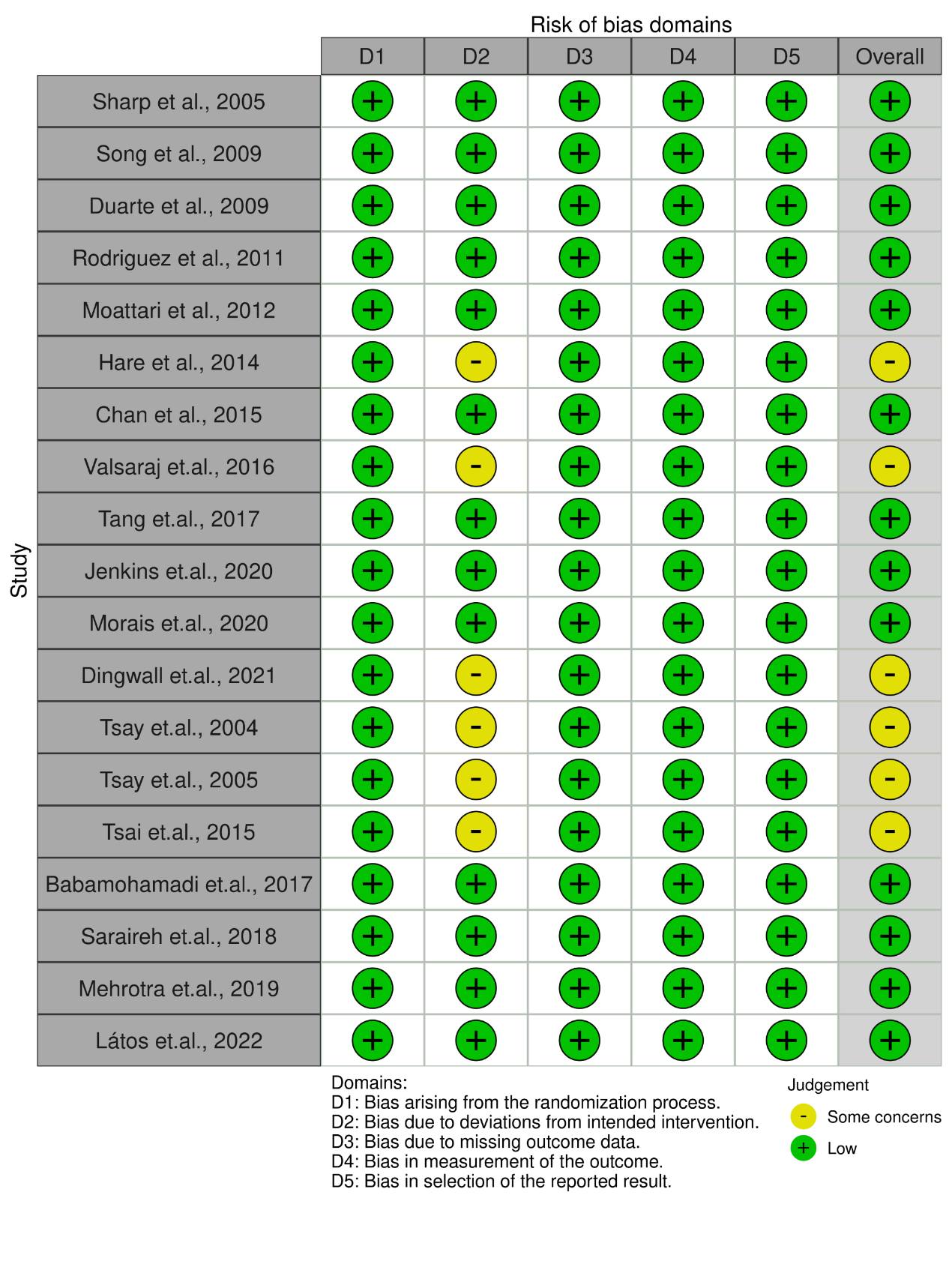
Figure S1. Risk of bias assessment of included studies using the revised Cochrane risk of bias tool**

**
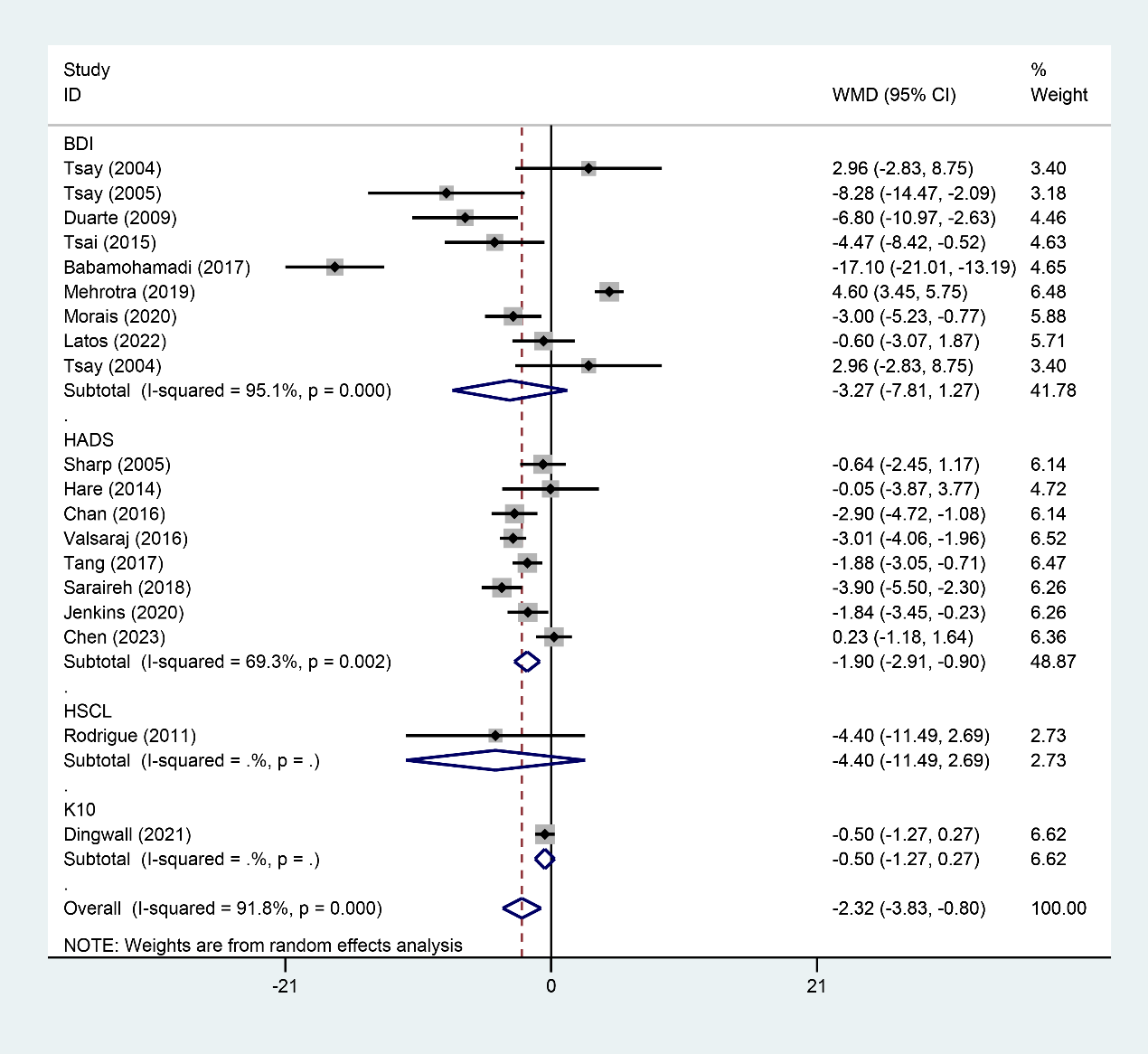
**

**Figure S2. Forest plot of subgroup analyses on changes of depression.**

**
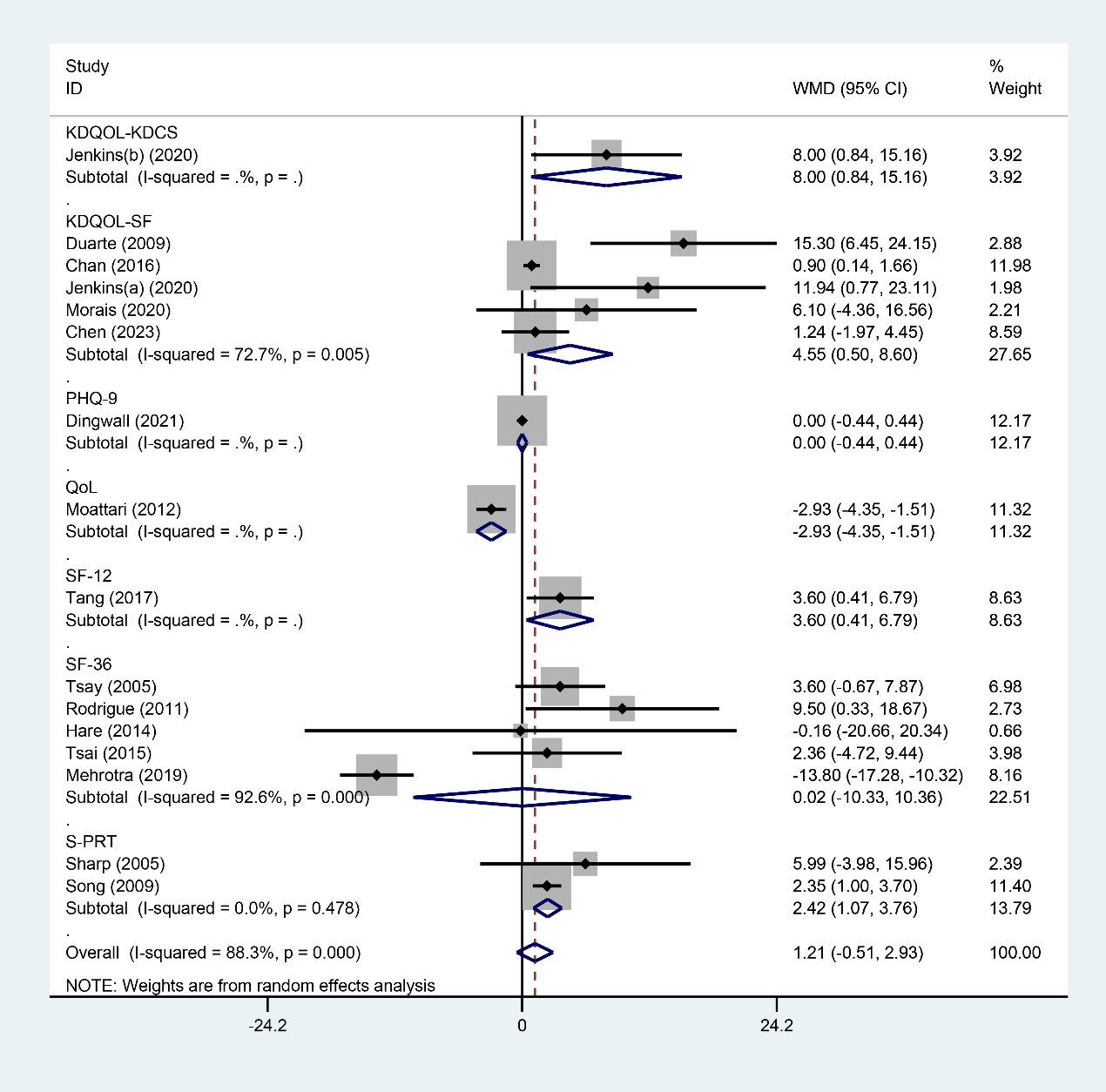
**

**Figure S3. Forest plot of changes of subgroup analyses on quality of life.**

**
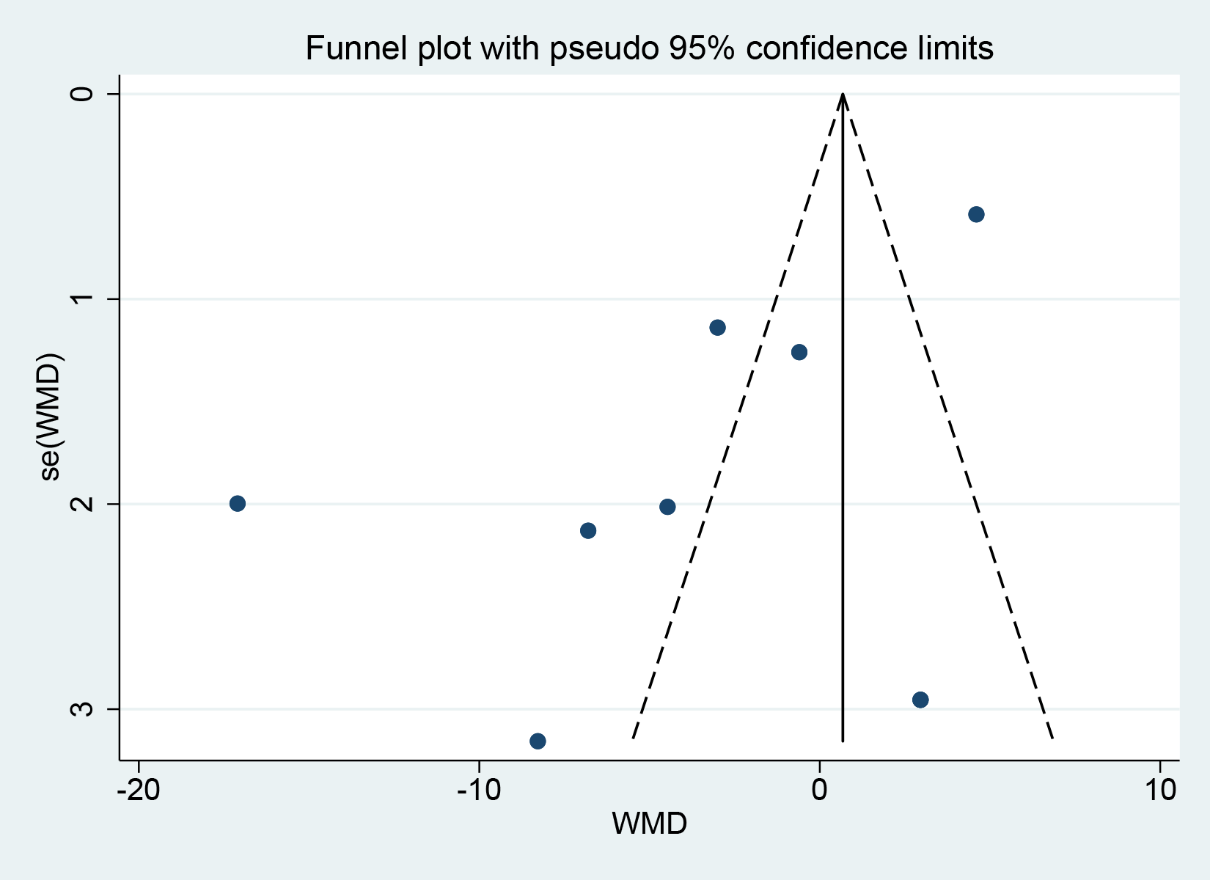
**

**Figure S4. Funnel plot for pooled results of included studies on changes of BDI**

**
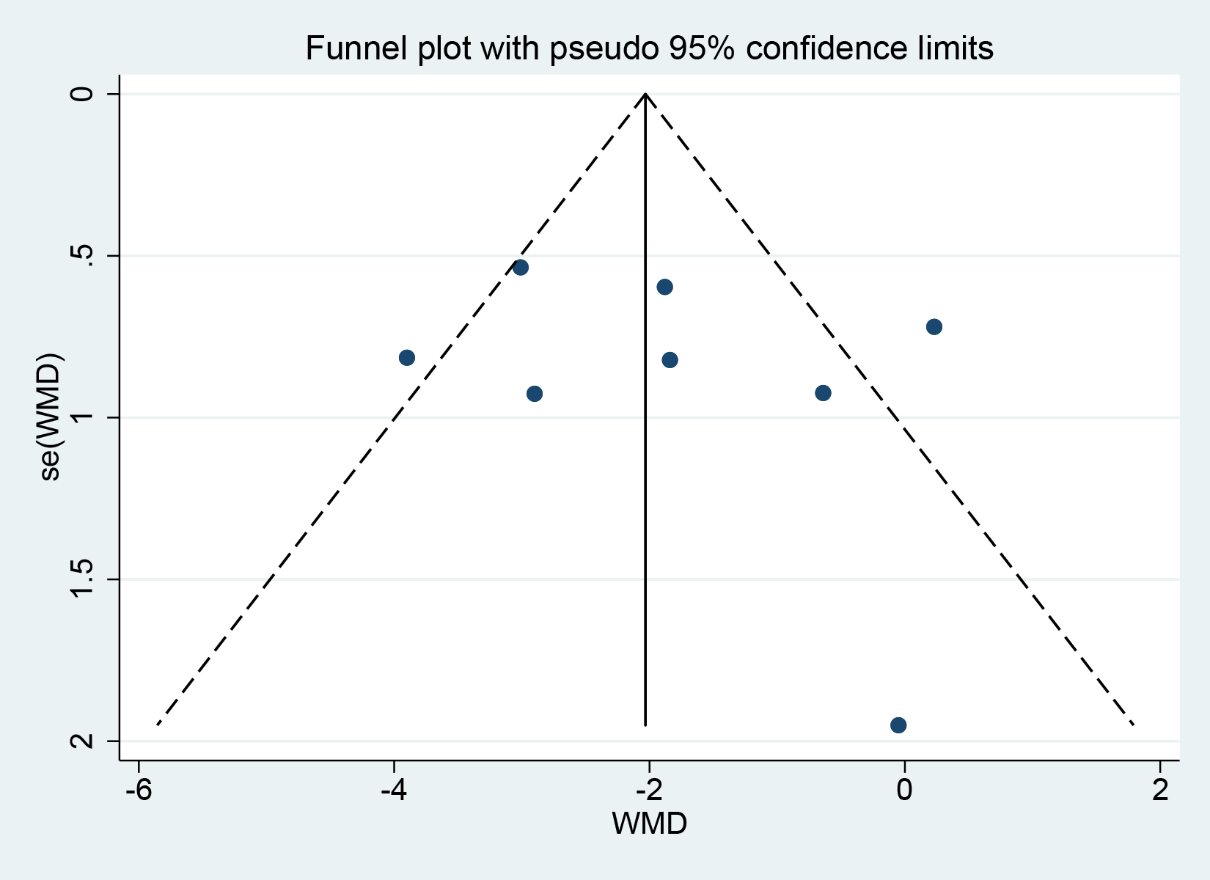
**

**Figure S5. Funnel plot for pooled results of included studies on changes of HADS**

**
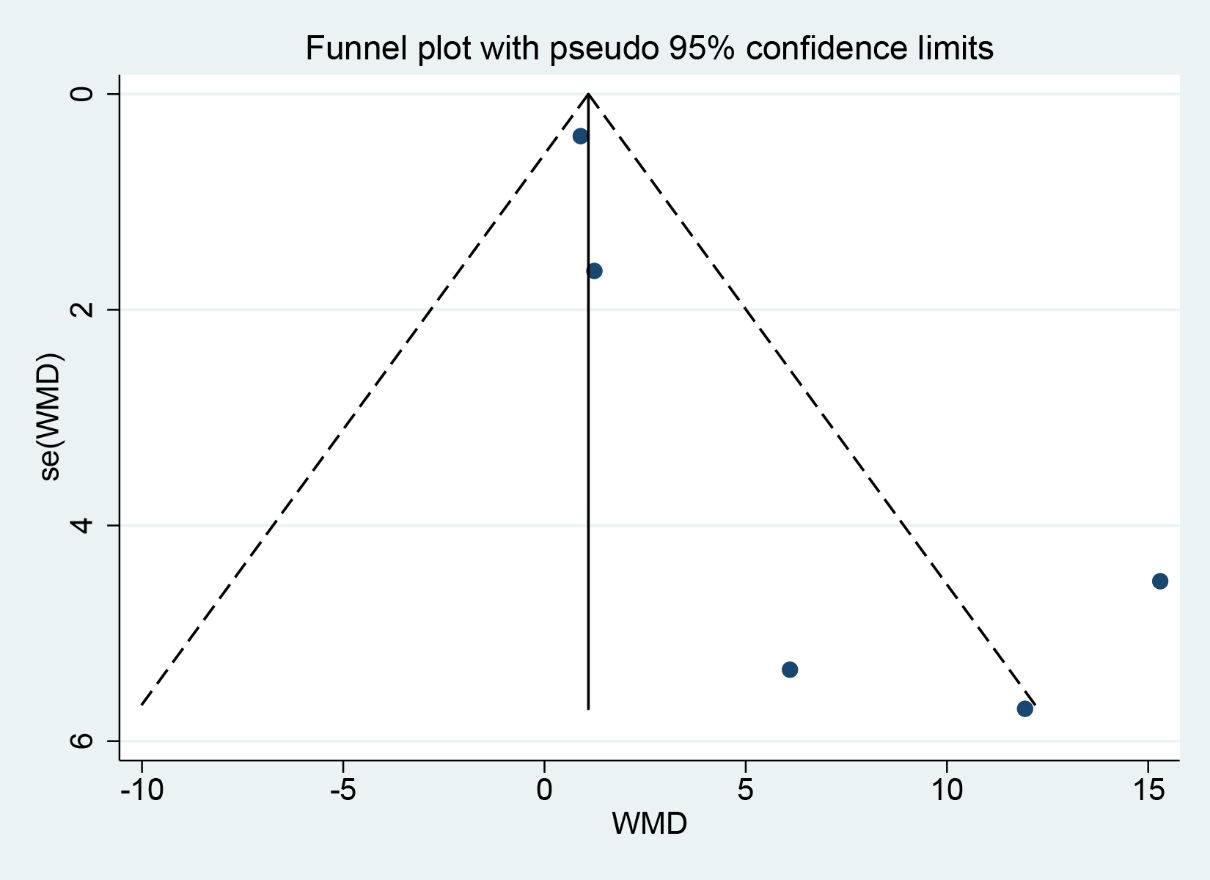
**

**Figure S6. Funnel plot for pooled results of included studies on changes of** **KDQOL-SF**

**
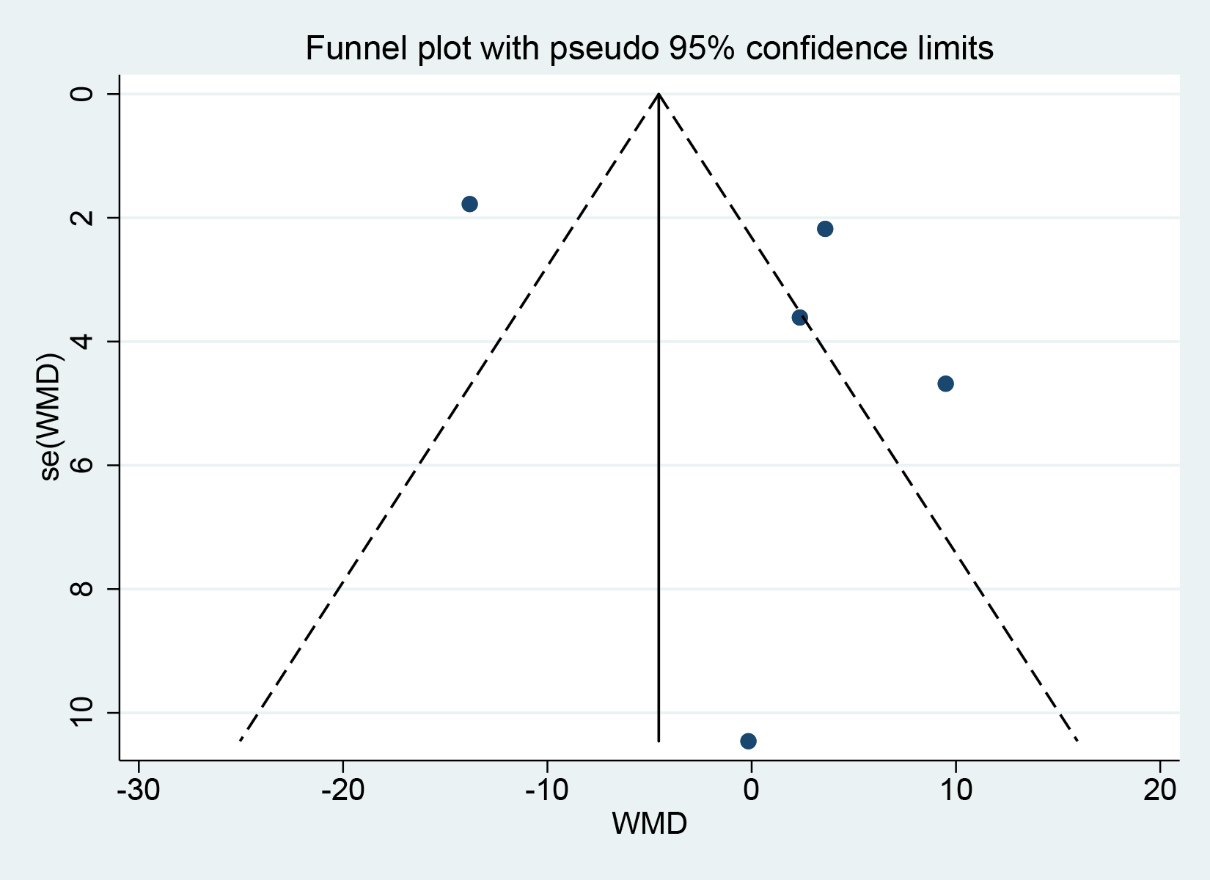
**

**Figure S7. Funnel plot for pooled results of included studies on changes of SF-36**

**
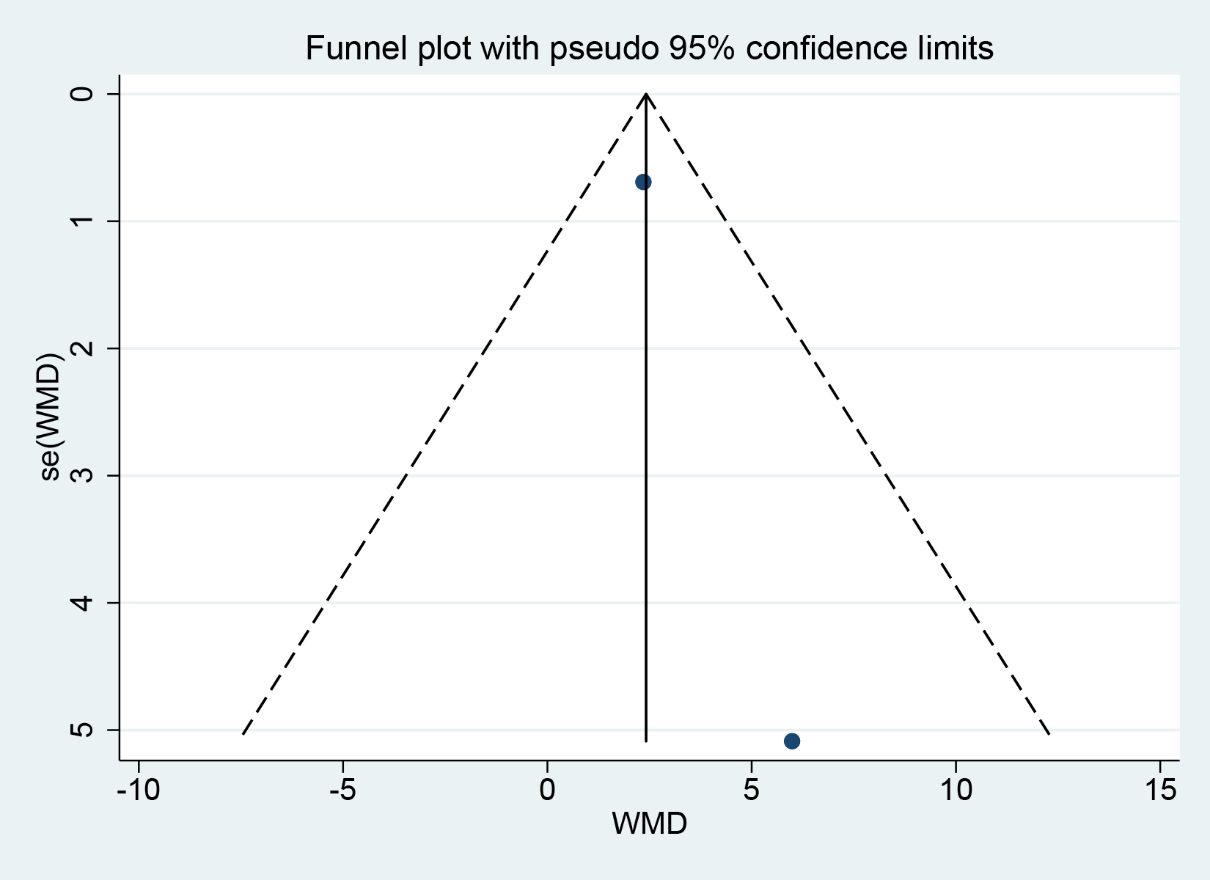
**

**Figure S8. Funnel plot for pooled results of included studies on changes of SPRT**

**
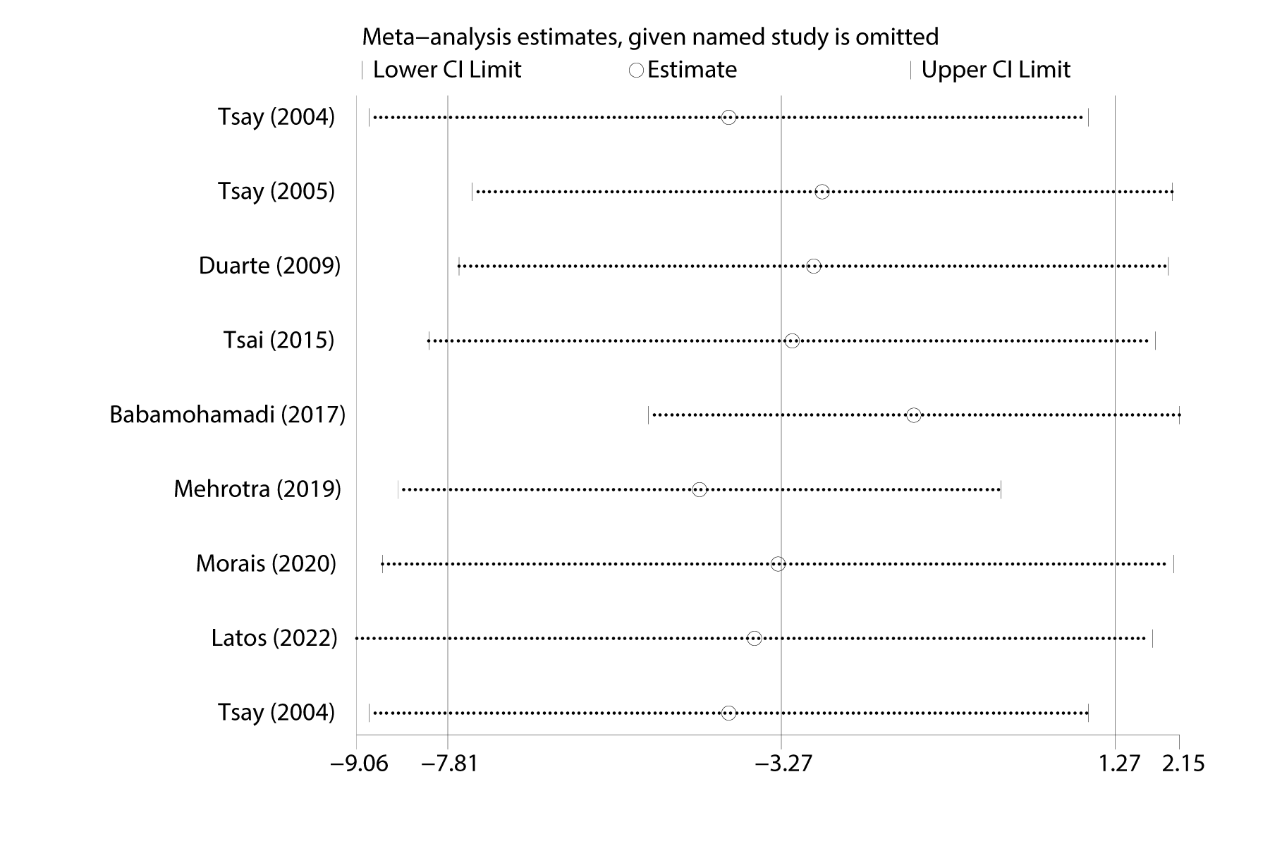
**

**Figure S9. Results of sensitivity analysis for pooled results of included studies on changes of BDI**

**
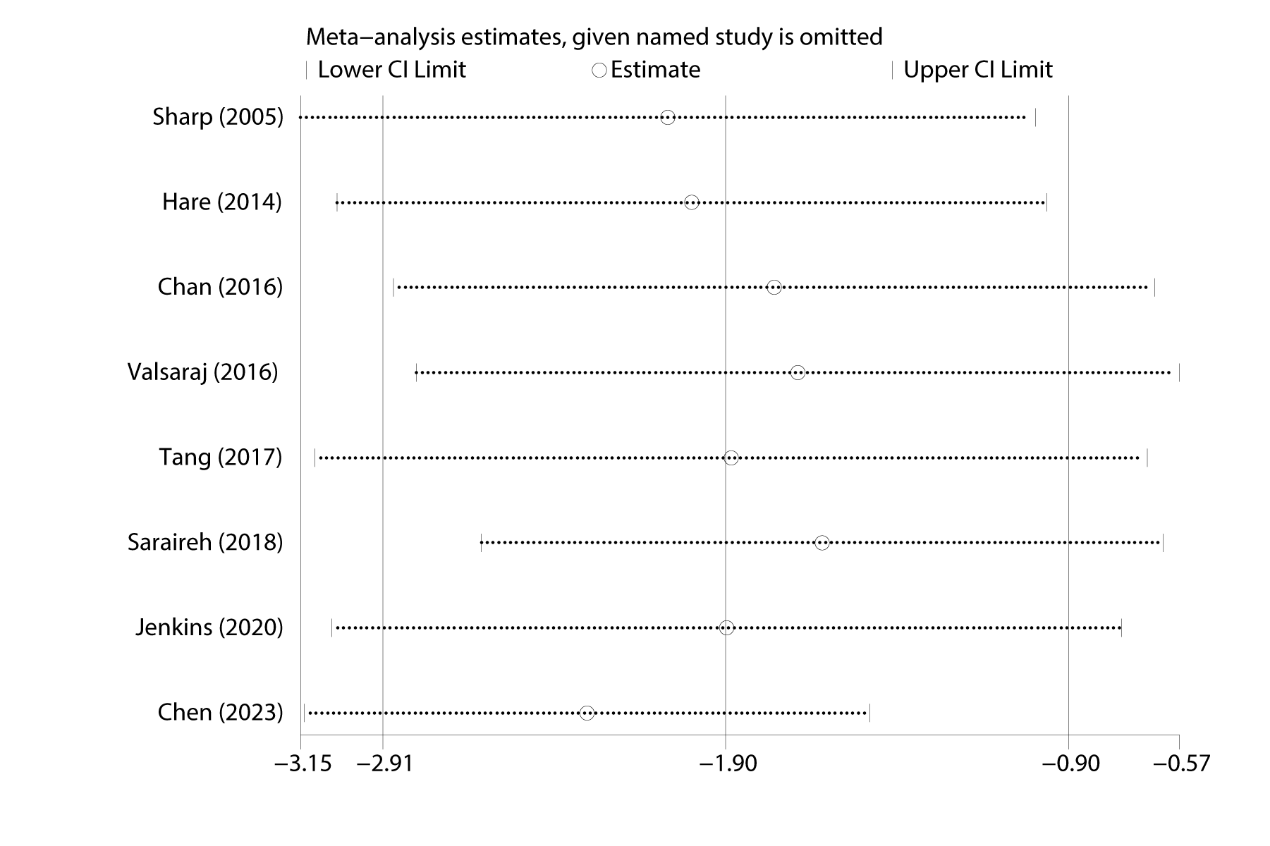
**

**Figure S10. Results of sensitivity analysis for pooled results of included studies on changes of HADS**

**
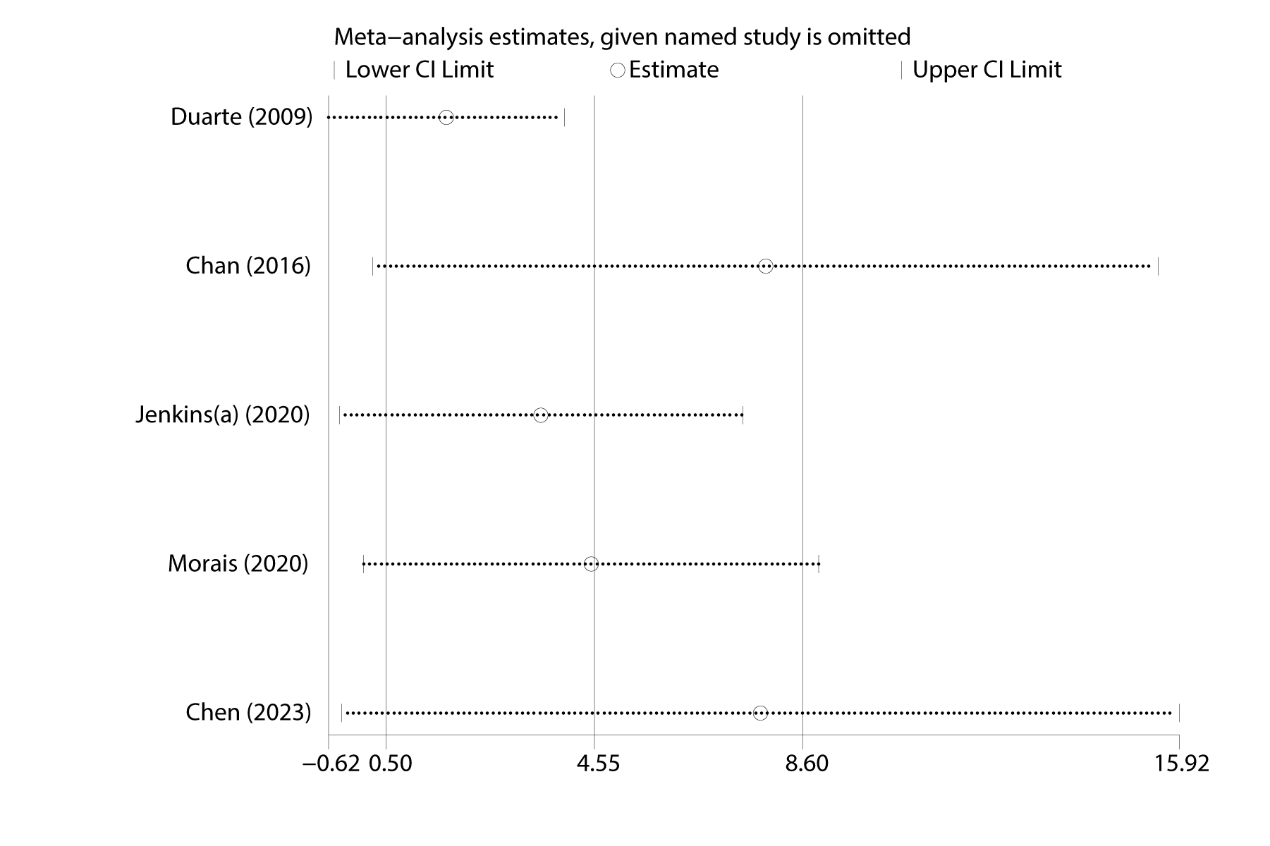
**

**Figure S11. Results of sensitivity analysis for pooled results of included studies on changes of KDQOL-SF**

**
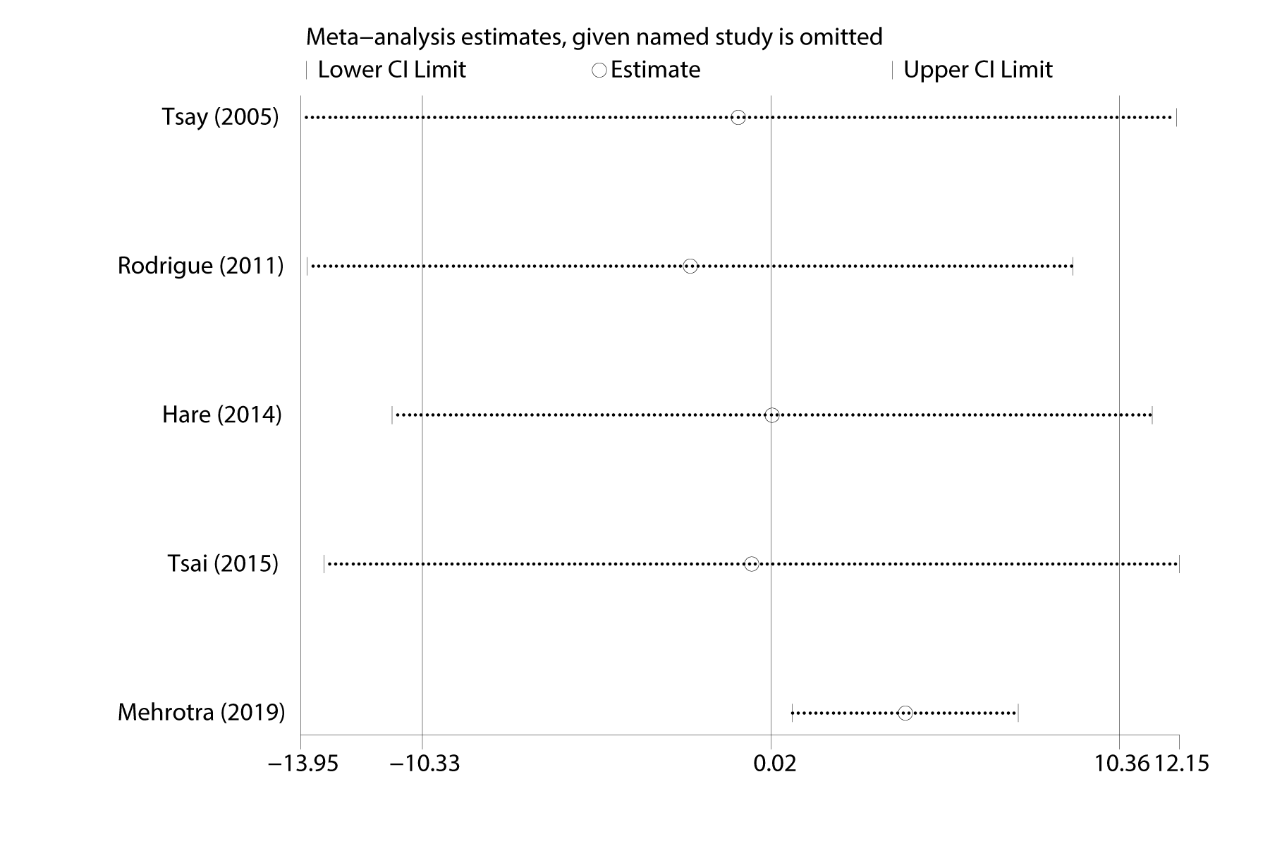
**

**Figure S12. Results of sensitivity analysis for pooled results of included studies on changes of SF-36**

**
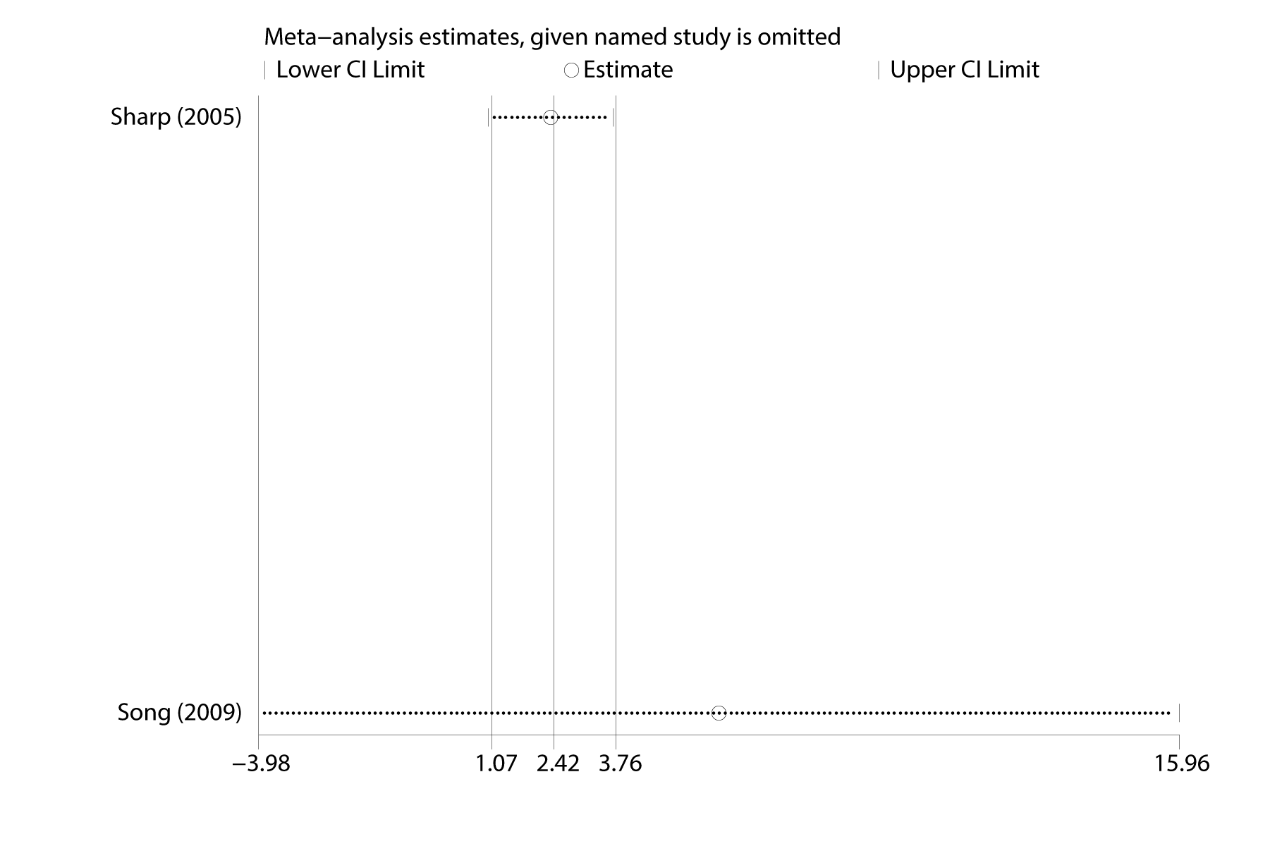
**

**Figure S13. Results of sensitivity analysis for pooled results of included studies on changes of SPRT**
